# The Correlation between Mandibular Arch Shape and Vertical Skeletal Pattern

**DOI:** 10.3390/medicina59111926

**Published:** 2023-10-31

**Authors:** Domenico Ciavarella, Mauro Lorusso, Carlotta Fanelli, Angela Pia Cazzolla, Marta Maci, Donatella Ferrara, Lorenzo Lo Muzio, Michele Tepedino

**Affiliations:** 1 Department of Clinical and Experimental Medicine, Dental School of Foggia, University of Foggia, Via Rovelli 50, 71122 Foggia, Italy; domenico.ciavarella@unifg.it (D.C.); carlotta.fanelli@unifg.it (C.F.); angelapia.cazzolla@unifg.it (A.P.C.); marta.maci@unifg.it (M.M.); donatella.ferrara@unifg.it (D.F.); lorenzo.lomuzio@unifg.it (L.L.M.); 2 Department of Biotechnological and Applied Clinical Sciences, Dental School of L’Aquila, University of L’Aquila, 67100 L’Aquila, Italy; m.tepedino@hotmail.it

**Keywords:** arch form, growth evaluation, orthodontic treatment, vertical growth

## Abstract

*Background and Objectives*: The aim of this study was to evaluate the correlation between the mandibular arch shape and the vertical skeletal pattern in growing patients. *Materials and Methods*: A total of 73 Caucasian patients (33 males and 40 females; mean age 9.4) were retrospectively enrolled from a pool of patients treated in chronological order at the Department of Orthodontics, University of Foggia, Italy, from April 2018 to December 2021. Each patient received a laterolateral radiograph and a digital scan of the dental arch. Eight cephalometric parameters (lower gonial angle, intermaxillary angle, divergence angle, Wits index, Jarabak ratio, OP-MP angle, PP-OP angle, and ANB) and five dental measurements (posterior mandibular arch width, anterior mandibular arch width, mandibular occlusal angle, posterior width on distobuccal molar cusps, and molar angle) were analyzed and then compared. A Spearman’s rho correlation test between the cephalometric measurements and the dental measurements was performed. Statistical significance was set at *p* < 0.05. *Results*: A negative statistically significant correlation was found between the Jarabak ratio and the intermolar angle; a statistically significant correlation was also observed between the Wits index, the posterior mandibular width, and the occlusal mandibular angle; the ANB angle and the occlusal mandibular angle; the intermaxillary angle (PP-PM) and the mandibular occlusal angle, posterior mandibular width on the disto-vestibular cusp, and the intermolar angle; and the OP-MP angle and mandibular occlusal angle and the posterior mandibular width on the disto-vestibular cusp. *Conclusions*: The mandibular arch form may be related to certain predisposing features in craniofacial morphology, such as jaw divergence, the Jarabak ratio, and the intermaxillary angle.

## 1. Introduction

Dental arch growth is a process involving multiple factors. Arch development depends on both genetic and epigenetic components, including muscular, functional, and local factors [1]. Jaw growth occurs through a dual mechanism: the direct growth of the basal bone and the indirect displacement caused by the growth of the cranial bones. The dentoalveolar component is able to implement compensatory mechanisms to change the spatial relationship between the maxilla and the mandible, which can be intercepted and identified [2]. Overall, growth depends on a complementary action between synchondrosis and sutures activities, cortical bone remodeling, and displacement [3]. The combination of all these processes ultimately results in a unique facial morphology. Arguably, one of the most important aspects to consider during orthodontic diagnosis and treatment planning is how those growth processes combine on the vertical plane. Moreover, the sum of the facial skeletal growth and the dentoalveolar compensatory mechanism determines the spatial orientation of the occlusal plane, and the nature of the interaction between those two components is not trivial. According to Schudy [4], by taking the anterior cranial base (S-N) as a reference, it is possible to study the mandibular plane orientation (Go-Me) by measuring the divergence angle (SN^GoMe). In addition, the facial divergence also seems to be related to the transverse dimension: Nasby et al. [5] observed increased mandibular molar diameters and an increased length of the maxillary and mandibular dental arches in patients with reduced SN-MP. Dolichofacial subjects with increased SN-MP showed reduced maxillary and mandibular transverse diameters, while brachyfacial subjects (reduced SN-MP) showed increased transverse diameters [6]. However, El Haje et al. [7] did not observe significant correlations between mandibular arch shape and transverse and vertical diameters in a sample of patients aged 15 to 19 years. Therefore, there seems to be an interaction between the vertical biotype and the dental arch form, as observed by Ocak et al. [8], and understanding the morphological differences associated with vertical facial divergence patterns is critical for providing the correct therapeutic treatment. Some authors have analyzed the correlation between the maxillary arch shape and the vertical growth pattern in a specific population, thus highlighting the likely influence of genes that characterize and affect facial growth [9]. Morphological differences between skeletal facial types are already evident around 5–6 years of age [10]; later, the alternate periods of intense and less intense growth, bones remodeling by apposition and resorption, the alveolar processes’ development and teeth eruption through continuous changes in facial skeletal proportions create different relationships in the different facial biotypes [3]. Furthermore, different authors have analyzed the correlation between the sagittal and vertical skeletal pattern and the development of class II and III malocclusions [11]. Therefore, understanding the relationship between those components could provide the instruments to more precisely predict the patient’s growth pattern and future development. Moreover, even when growth is already complete and the morphological features have already been established, knowing the relationship between facial type and arch form could help to achieve more stable results by respecting the patient’s biological arch form during fixed orthodontic treatment. During development stages, the eruption of deciduous teeth and later, permanent teeth, leads to an increase in the three-dimensional skeletal component, so the sagittal growth of the jaw is influenced by vertical and transverse growth, and bone remodeling should provide a balance between the upper third and the middle and lower thirds of the face [12]. Moreover, the sagittal pattern is influenced by the mandibular rotational growth pattern through glenoid fossae remodeling and masticatory cycle neuro-muscular activity, and thus from the vertical pattern, which determines the relationship between the jaws. Indeed, Chae et al. [13] observed a different position of the condyle in the glenoid fossa in hyperdivergent and hypodivergent patients with class II malocclusion. To the authors’ knowledge, there are no studies analyzing the relationship between sagittal pattern and mandibular arch form, which still remains unclear.

Therefore, the aim of this study was to evaluate the correlation between the mandibular arch shape and the vertical skeletal pattern in growing patients. Moreover, the relationship between arch form and sagittal growth pattern was evaluated. The null hypothesis is that no correlation exists between the vertical and sagittal skeletal pattern and the mandibular arch shape.

## 2. Materials and Methods

This study was reported following the Strengthening The Reporting of Observational Studies in Epidemiology (STROBE) guidelines for observational studies [14].

All the procedures of this research protocol have adhered to the Declaration of Helsinki and have been approved by the Ethics Committee of the University of Foggia (Approval no.43/CE/2019). The records were retrieved retrospectively, were analyzed anonymously, and the patients signed a written informed consent. The inclusion and exclusion criteria are listed in Table 1. Skeletal age was determined by the cervical vertebral maturation (CVM) method using lateral cephalometric radiography [15]. The sociodemographic characteristics of the sample are listed in Table 1.

A power analysis [16] (G*Power 3.1.9.2, Franz Faul, Universitat Kiel, Kiel, Germany) revealed that to detect a large effect size of 0.4 with a linear multiple regression, with an α error prob of 0.05 and a power (1-β error prob) of 0.95, 71 subjects would be required.

A total of 73 untreated Caucasian patients (33 males and 40 females), with a mean age of 9.4, were retrospectively enrolled in the present study from a pool of patients treated in chronological order at the Department of Orthodontics, University of Foggia, Italy, from April 2018 to December 2021.

All the following documentation was collected for each patient:

- Orthopantomography and lateral cephalogram;
- Photographs: intraoral and extraoral photos;
- Digital scans of the dental arches.

### 2.1. Cephalometric Analysis

**Figure 1:**
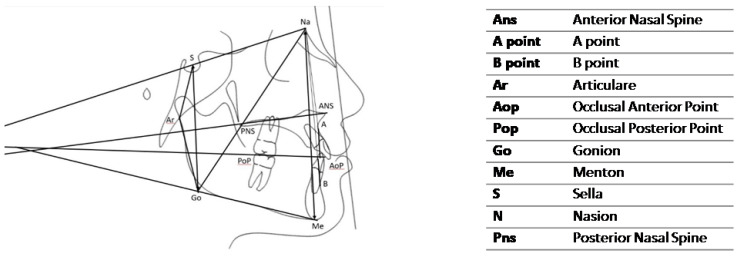
Cephalometric landmarks and reference lines.

**Figure 2:**
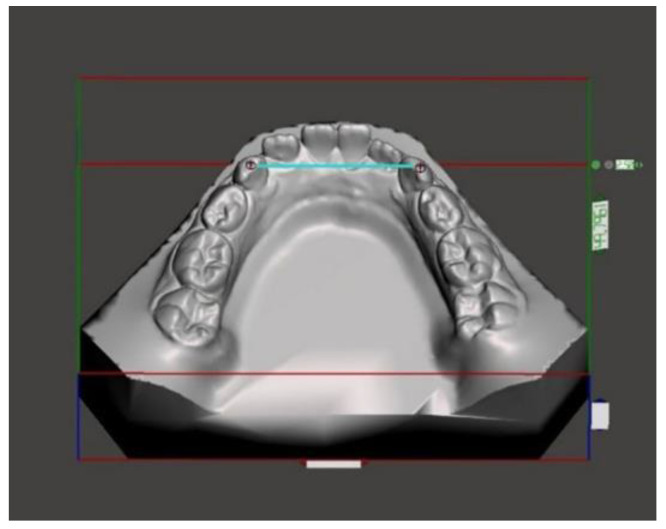
Anterior mandibular width.

**Figure 3:**
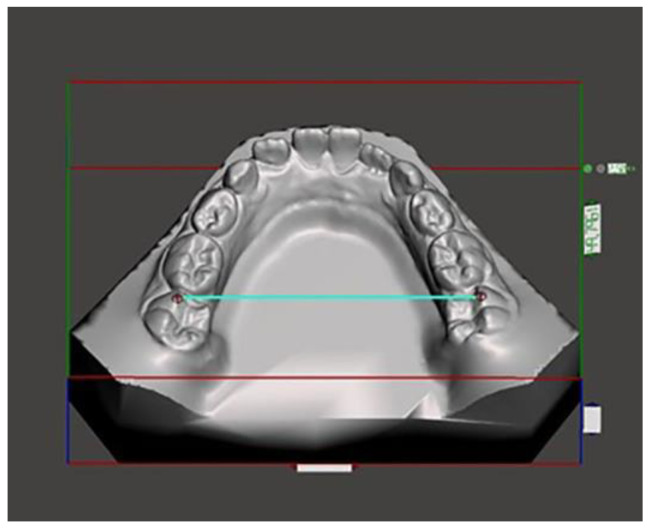
Posterior mandibular width.

**Figure 4:**
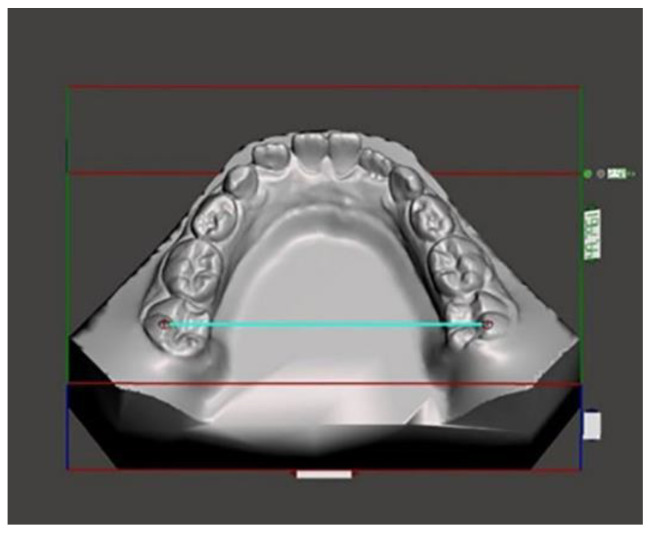
Posterior mandibular width measured on the disto-vestibular cusp of the first molar.

**Figure 5:**
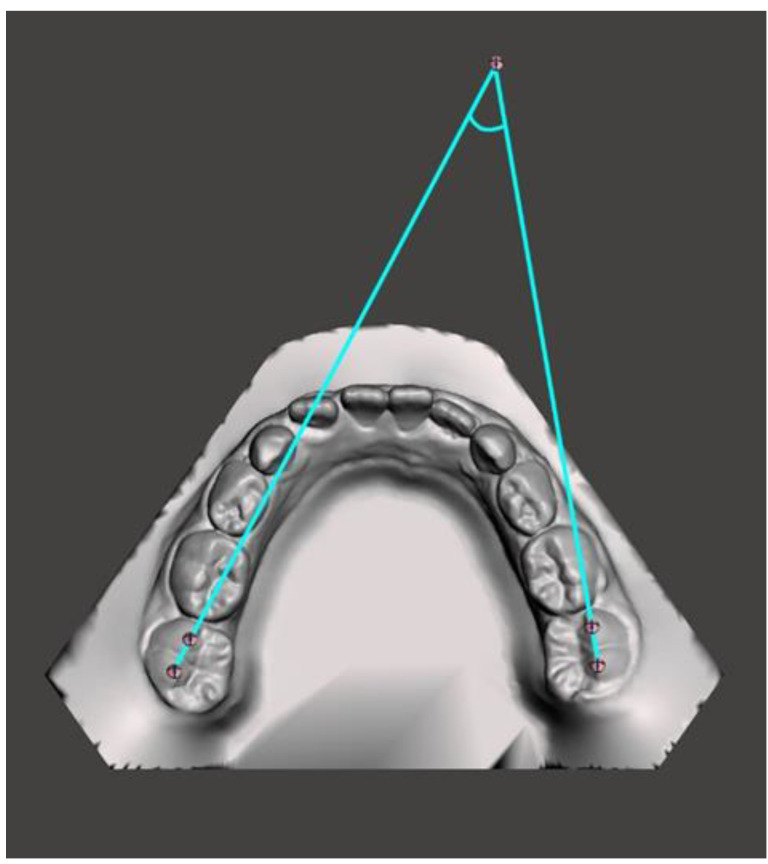
Intermolar angle.

**Figure 6:**
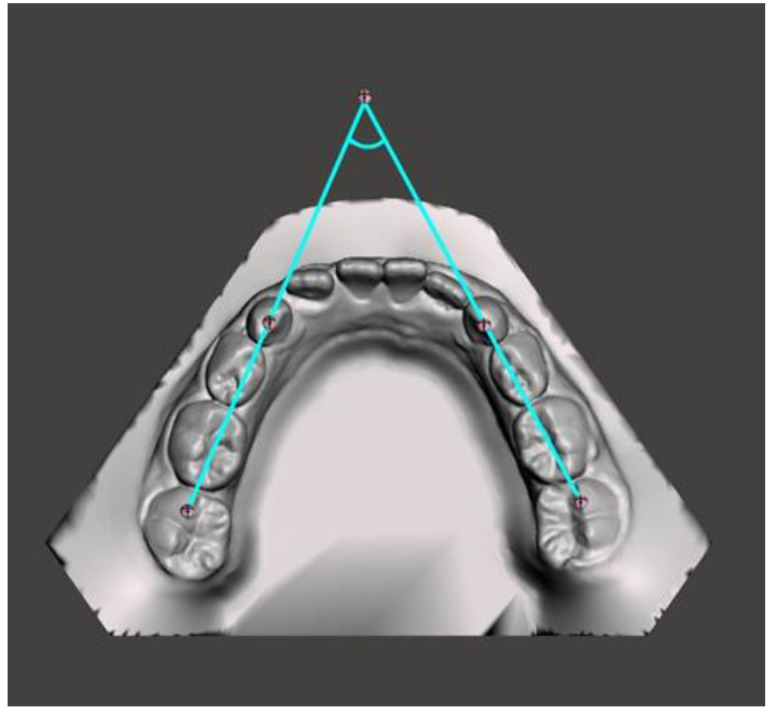
Mandibular occlusal angle.

Lateral head films (Gendex GXDP-700) were obtained, with the patient’s head positioned in a cephalostat, in centric occlusion, with adequate visualization of the reference structures, and without appreciable head rotation. All the lateral radiographs were captured by the same technician and on the same machine in the same radiology department. A cephalometric analysis was performed on the lateral cephalograms [17,18]. The following cephalometric skeletal variables were analyzed: SN-MP, PP-MP, OP-MP, PP-OP, N-GoMe, S-Go/N-Me, ANB, and Wits index. The landmarks and reference lines used in the cephalometric analysis were presented in Figure 1 and described in Table 2. The dental measurements are listed in Table 3 and described in Figure 2, Figure 3, Figure 4, Figure 5 and Figure 6. The linear and angular measurements, indicated in Table 2, were performed for each mandibular arch digital scan. To reduce the errors in the method, the cephalometric analyses were performed by a trained examiner, and all measurements were conducted twice by the same operator.

### 2.2. Statistical Analysis

Data distribution analysis was conducted using the Shapiro–Wilk normality test (Table 4). Descriptive statistics were also obtained (Table 4). Because the variables failed the normality test, a Spearman rho test was used to analyze the correlation between the cephalometric measurements and the arch width measurements. The significance index was set to *p* < 0.05. The data were analyzed using GraphPad Prism software 6.0 (GraphPad Prism Software, San Diego, CA, USA).

To reduce random errors, the cephalometric and dental measurements were calculated twice. The random error of each measurement was calculated using Dahlberg’s formula (*S* = ∑ *d*^2^/2*N*), where *d* is the difference between the first and second measurements and *N* the number of radiographs evaluated [19,20]. The random error ranged between 0.56 and 1.38 mm for the linear measurements and between 0.78 and 0.95 degrees for the angular measurements.

## 3. Results

The results of the statistical analysis (Table 5) were divided into two groups, according to the rho coefficient.

A significant good correlation (rho > 0.3) was observed between:

- The Wits index and the posterior mandibular width (rho = 0.430, *p* < 0.01);
- The Wits index and the mandibular occlusal angle (rho = 0.543, *p* < 0.01);
- The intermaxillary angle (PP-PM) and the mandibular occlusal angle (rho = 0.416, *p* < 0.01);
- The intermaxillary angle (PP-PM) and the posterior mandibular width on the disto-vestibular cusp (rho = 0.358, *p* < 0.01);
- The Jaraback ratio and the intermolar angle (rho = −0.396, *p* < 0.01).

A significant but weak correlation (rho < 0.3) was observed between:

- The ANB angle and the mandibular occlusal angle (rho = 0.245, *p* < 0.05);
- The intermaxillary angle (PP-PM) and the intermolar angle (rho = 0.279, *p* < 0.05);
- Shudy’s angle and the mandibular occlusal angle (rho = 0.287, *p* < 0.05);
- Shudy’s angle and the posterior mandibular width on the disto-vestibular cusp (rho = 0.281, *p* < 0.05).
- Therefore, the null hypothesis was rejected.

## 4. Discussion

The study’s objective was to assess the correlation between mandibular arch shape and the vertical and sagittal skeletal pattern in growing patients. This evaluation aimed to emphasize early clinical signs detected on mandibular arches for diagnosing nonphysiological occlusal conditions associated with altered growth patterns. Furthermore, the study sought to supply supplementary data to assist in the selection of the arch shape during straight-wire treatment.

Craniofacial growth follows a defined development timing [21]. The transverse growth is completed first, followed by the sagittal and the vertical growth. In a sample of growing patients without previous orthodontic treatment, Wagner et al. [6] reported that while maxillary transverse growth reached a plateau around 14 years of age, the mandibular transverse skeletal width continued to increase in subjects with a normal or reduced mandibular plane angle. On the contrary, the mandibular skeletal width showed a plateau in subjects with an increased mandibular plane angle. In other words, differences in transverse mandibular growth was observed in patients with a different vertical pattern.

Various studies have analyzed the correlation between vertical pattern, using mandibular plane angle, and arch form in class II patients [22,23,24,25]. Grippaudo et al. [26] found no statistically significant difference in the mandibular arch form between hyper-, normo-, and hypodivergent class II malocclusion patients. On the other hand, the relationship between the sagittal plane and mandibular arch form has never been investigated, although the sagittal plane shows a complex interaction with the vertical and transversal plane. The results obtained from the present study showed that as the ANB angle and Wits index increase, the mandibular occlusal angle increases, i.e., in patients with class II malocclusion, there is an increase in posterior arch width; the mandible, therefore, will have an ovoid shape (U-shape).

In the present study, a significant correlation was observed between the intermaxillary angle (PP-PM) and the mandibular occlusal angle, as well as between the posterior mandibular width on the disto-vestibular cusp and the intermolar angle. Hyperdivergent subjects showed an increase in the posterior mandibular width resulting in a V-shaped mandibular arch. Anwar et al. [23] reported that mandibular intermolar width showed a progressive increase in subjects from hyperdivergent vertical skeletal patterns towards normo and hypodivergent. On the contrary, Forster et al. [27] found no statistically significant correlation between mandibular plane angle and intermolar distance. Hwang et al. [28] found no statistically significant differences in mandibular intermolar distance and molar inclination between hypodivergent, normodivergent and hyperdivergent adult groups. However, Grippaudo et al. [26] reported no differences in intercanine and intermolar distance between different vertical growth pattern patients. Additionally, they found a prevalence of a mandibular V-shape arch in subjects with reduced mandibular plane angle and a prevalence of mandibular ovoid-shape arches in patients with increased mandibular plane angles. Likely, in hyperdivergent subjects, the transverse growth was dislocated in the mandibular posterior region, as occlusal forces were focused in the posterior sectors. A significant correlation of the PM-PO angle with the mandibular occlusal angle and the posterior mandibular width was also observed in the present study. In patients with an increased PM-PO angle, an increased posterior mandibular width was observed. It is unclear whether the molars were able to create compensation through a different inclination as the vertical growth pattern changes. Some authors [6,27,28] observed that subjects with an increased vertical dimension had a vestibular inclination of the posterior dental group, while subjects with a decreased vertical dimension showed a lingual inclination of the posterior dental group. Likely, in subjects with hypodivergent or normodivergent growth patterns, mandibular molars compensated through a lingual inclination to maintain an adequate ratio between the maxillary and the mandibular intermolar distance. Similarly, a negative correlation between the Jarabak ratio and the intermolar angle was observed, so that as the ratio decreases, an increase in the intermolar angle was observed. Again, this result confirmed that in hyperdivergent patients, there was an increase in the posterior mandibular width. On the contrary, Anwar et al. [23] observed an increase in the posterior mandibular width from hyper- to normo- to hypodivergent. Furthermore, the authors highlighted that as skeletal divergence increased, as evaluated through the intermaxillary divergence angle (PP-MP), the mandibular dental arch tended to a tapered shape (V-shaped), with a narrower anterior mandibular arch and a wider posterior arch. In patients with divergence angle reduction and therefore, with a reduced jaw divergence, the mandibular dental arch presented a more ovoid (U) shape. This difference was detected through the mandibular occlusal angle variations.

A possible role of the musculature should be taken into consideration when studying the relationship between the transverse arch dimensions and the vertical skeletal pattern. Few studies associating facial musculature with craniofacial growth have reported that muscle hyperfunction was evident in hypodivergent subjects [29,30]. Increased loading due to hyperfunction of the masticatory muscles can lead to increased sutural growth and bone apposition, resulting in increased transverse growth and wider bone bases for the dental arches [29]. On the contrary, in hyperdivergent subjects, there was a narrower dental arch, a greater palatal height, and a lower bite force [27,28]. However, according to Kiliaridis et al. [29], the epigenetic influence of the masticatory muscles, in their role as force-generating elements on craniofacial growth, may be valid in the presence of the increased muscle activity, but this is not necessarily true when this activity is reduced.

According to the results shown in the present study, in hyperdivergent subjects treated with fixed multibracket therapy, a tapered mandibular arch should be used; on the contrary, in hypodivergent subjects, an ovoid arch shape should be used. The mandibular arch shape is determined by the interaction between functional capabilities, masticatory muscles action [31], and basal bone shape. Therapeutic modifications should respect these factors in order to perform only necessary modifications that lead to stable results. It is unclear whether preformed wires are able to fit every patient’s arch shape. According to Mughal et al. [32], commercial archwires are generally larger, in particular in regards to the intercanine distance. The use of these preformed arches should be avoided, since it could compromise treatment stability and increase the risk of relapse. Archwire selection should consider the vertical growth pattern and malocclusion type [33]; to achieve good long-term stability, arches should be shaped by the clinician at the chairside to fit the patient’s anatomy. 

### Limitation of the Study

One limitation of this study is the result of the retrospective nature of patient recruitment, although care was taken to avoid any selection bias, thanks to the use of a rigid chronological criterion. A further limitation is related to the bi-dimensional characteristics of the cephalometric exam used. Due to the retrospective nature of this study, it is difficult to determine any other unanalyzed variables that might have influenced the relationship between the mandibular arch measurement and the cephalometric parameters. As the untreated subjects were not recruited from a population sample, but from a university dental clinic, some inherent bias might be possible.

## 5. Conclusions

The findings of the present study can be summarized as follows:

- Increased jaw divergence correlates with an increased posterior width of the mandibular dental arch, measured at the disto-buccal cusp of the lower first molar;
- Increased jaw divergence is associated with a tapered (V-shaped) mandibular dental arch shape, with a narrower anterior part and a wider posterior part.

The distal positioning of the mandible (skeletal class II) is associated with a tapered (V-shaped) mandibular dental arch shape and an increase in the posterior mandibular dental arch width.

## Figures and Tables

**Table 1:** (**A**) Inclusion and exclusion criteria. (**B**) Sociodemographic characteristics of the sample.

(**A**)	
Inclusion Criteria	Exclusion Criteria
Complete eruption of permanent incisors and first permanent molars;	Mono or bilateral cross-bite; Mono or bilateral scissor-bite;
Age between 8 and 10 years; 25° < SN-MP < 38°;	Patients with complete permanent teeth;
Lateral cephalogram performed with the same cephalostat;	Skeletal malformations and destructive caries;
Skeletal age between (CS2 and CS3), according to the CVM method;	Previous cervical trauma;
Absence of temporomandibular joint disorders;	Patients with previous orthodontic treatment;
No maxillofacial and airway surgery.	First molar rotated.
(**B**)	
SAMPLE	*n* = 77
MALES	33 (45.2%)
FEMALES	40 (54.8%)
AGE	9.4 ± 0.8
FISHER TEST FOR GENDER	*p* < 0.001

**Table 2:** Skeletal cephalometric measurements.

Skeletal Measurements	
Measurement	Description
SN-MP	Angle between the sella-nasion (SN) line and the mandibular plane (MP)
PP-MP	Angle between the palatal plane (PP) and the mandibular plane (MP)
OP-MP	Shudy’s angle: the angle between the occlusal plane (OP) and the mandibular plane (MP)
PP-OP	Angle between the palatal plane and the occlusal plane (OP)
LOWER GONIAL ANGLE	Angle between the gonion-nasion (GoNa) line and the gonion-menton (GoMe) line
JARABACK RATIO	Ratio between posterior facial height and anterior facial height
Wits Index	Distance between the orthogonal projections of A and B points on the occlusal plane
ANB	Angle between the nasion-A line and nasion-B line, obtained by subtracting the SNB angle from SNA angle.

**Table 3:** Dental measurements.

Dental Measurements	
Measurement	Description
ANTERIOR MANDIBULAR WIDTH	Linear distance between the mandibular canine cusps
POSTERIOR MANDIBULAR WIDTH	Linear distance between the mesiobuccal cusps of the first permanent molars
POSTERIOR MANDIBULAR WIDTH ON DISTOBUCCAL MOLAR CUSPS	Linear distance between the distobuccal cusps of the first permanent molars
INTERMOLAR ANGLE	Angle between the line that crosses the left first permanent molar mesiobuccal and distobuccal cusps and the line that crosses the right first permanent molar mesiobuccal and distobuccal cusps
MANDIBULAR OCCLUSAL ANGLE	Angle between the line that crosses the first left permanent molar mesiobuccal cusp and the left canine cusp and the line that crosses the first right permanent molar mesiobuccal cusp and the right canine cusp

**Table 4:** Descriptive statistics and normality test.

Variables	Number of Values	Mean	Std.Dev	Median	Passed Normality Test
1	73	33.95	6.913	33.2	no
2	73	26.01	6.275	25.5	no
3	73	14.97	5.059	13.9	no
4	73	11.28	3.694	12.3	yes
5	73	79.5	5.56	80.2	no
6	73	65.17	5.681	66.2	no
7	73	1.352	3.953	1.2	no
8	73	2.745	1.781	2.5	yes
9	73	44.79	2.625	43	no
10	73	26.47	1.936	28	yes
11	73	46.87	4.427	45	no
12	73	47.41	2.992	48.3	yes
13	73	40.05	7.905	41	yes

Legend: 1: SN-MP ANGLE. 2: PP-MP ANGLE. 3: OP-MP ANGLE. 4: PP-OP ANGLE. 5: LOWER GONIAL ANGLE. 6: JARABACK RATIO. 7: WITS index. 8: ANB. 9: POSTERIOR MANDIBULAR WIDTH. 10: ANTERIOR MANDIBULAR WIDTH. 11: MANDIBULAR OCCLUSAL ANGLE. 12: POSTERIOR WIDTH ON DISTOBUCCAL CUSPS. 13: INTERMOLAR ANGLE.

**Table 5:** Spearman’ rho correlation test.

	SN-MP	PP-MP	PO-MP	PP-PO	LOWER GONIAL ANGLE	JARABACK RATIO	WITS Index	POPSTERIOR MANDIBULAR ARCH WIDTH	ANTERIOR MANDIBULAR ARCH WIDTH	MANDIBULAR OCCLUSAL ANGLE	POSTERIOR WIDTH ON DISTOBUCCAL MOLAR CUSPS	MOLAR ANGLE	ANB
SN-MP		0.811	0.758	0.333	0.438	−0.802	−0.175	−0.093	0.080	0.025	0.056	0.194	0.213
PP-MP	0.811		0.837	0.486	0.497	−0.625	−0.082	0.177	0.104	0.416 **	0.358 **	0.279 *	0.140
PO-MP	0.758	0.837		0.049	0.459	−0.649	0.019	0.106	0.074	0.287 *	0.281 *	0.209	−0.006
PP-PO	0.333	0.486	0.049		0.162	−0.170	−0.178	0.020	0.045	0.158	0.168	0.102	0.051
LOWER GONIAL ANGLE	0.438	0.497	0.459	0.162		−0.246	−0.438	−0.045	0.056	−0.194	−0.039	−0.152	−0.068
JARABACK RATIO	−0.802	−0.625	−0.649	−0.170	−0.246		0.199	−0.012	−0.037	0.067	−0.145	−0.396 **	−0.094
WITS Index	−0.175	−0.082	0.019	−0.178	−0.438	0.199		0.430 **	0.146	0.543 **	0.223	0.112	0.302
POSTERIOR MANDIBULAR ARCH WIDTH	−0.093	0.177	0.106	0.020	−0.045	−0.012	0.430		0.478	0.595	0.745	0.135	0.077
ANTERIOR MANDIBULAR ARCH WIDTH	0.080	0.104	0.074	0.045	0.056	−0.037	0.146	0.478		0.237	0.397	−0.100	0.146
MANDIBULAR OCCLUSAL ANGLE	0.025	0.416	0.287	0.158	−0.194	0.067	0.543	0.595	0.237		0.659	0.246	0.245
POSTERIOR WIDTH ON DISTOBUCCAL MOLAR CUSPS	0.056	0.358	0.281	0.168	−0.039	−0.145	0.223	0.745	0.397	0.659		0.403	0.111
MOLAR ANGLE	0.194	0.279	0.209	0.102	−0.152	−0.396	0.112	0.135	−0.100	0.246	0.403		0.042
ANB	0.213	0.140	−0.006	0.051	−0.068	−0.094	0.302	0.077	0.146	0.245 *	0.111	0.042	

* *p* < 0.05; ** *p* < 0.01.

## Data Availability

The data presented in this study are available on request from the corresponding author.
